# Imaging modalities to diagnose carotid artery stenosis: progress and prospect

**DOI:** 10.1186/s12938-019-0685-7

**Published:** 2019-05-28

**Authors:** Ashish Saxena, Eddie Yin Kwee Ng, Soo Teik Lim

**Affiliations:** 1 School of Mechanical and Aerospace Engineering, Nanyang Technological University, 50 Nanyang Ave, Block N3, Singapore, 639798 Singapore; 2 Department of Cardiology, National Heart Center Singapore, 5 Hospital Dr, Singapore, 169609 Singapore

**Keywords:** Carotid artery stenosis, Atherosclerosis, Diagnosis, Duplex ultrasound, Computed tomography angiography, Magnetic resonance angiography, Optical coherence tomography, Photoacoustic tomography, Infrared thermography

## Abstract

In the past few decades, imaging has been developed to a high level of sophistication. Improvements from one-dimension (1D) to 2D images, and from 2D images to 3D models, have revolutionized the field of imaging. This not only helps in diagnosing various critical and fatal diseases in the early stages but also contributes to making informed clinical decisions on the follow-up treatment profile. Carotid artery stenosis (CAS) may potentially cause debilitating stroke, and its accurate early detection is therefore important. In this paper, the technical development of various CAS diagnosis imaging modalities and its impact on the clinical efficacy is thoroughly reviewed. These imaging modalities include duplex ultrasound (DUS), computed tomography angiography (CTA) and magnetic resonance angiography (MRA). For each of the imaging modalities considered, imaging methodology (principle), critical imaging parameters, and the extent of imaging the vulnerable plaque are discussed. DUS is usually the initial recommended CAS diagnostic examination. However, for the therapeutic intervention, either MRA or CTA is recommended for confirmation, and for added information on intracranial cerebral circulation and aortic arch condition for procedural planning. Over the past few decades, the focus of CAS diagnosis has also shifted from pure stenosis quantification to plaque characterization. This has led to further advancement in the existing imaging tools and development of other potential imaging tools like Optical coherence tomography (OCT), photoacoustic tomography (PAT), and infrared (IR) thermography.

## Background

**Fig. 1:**
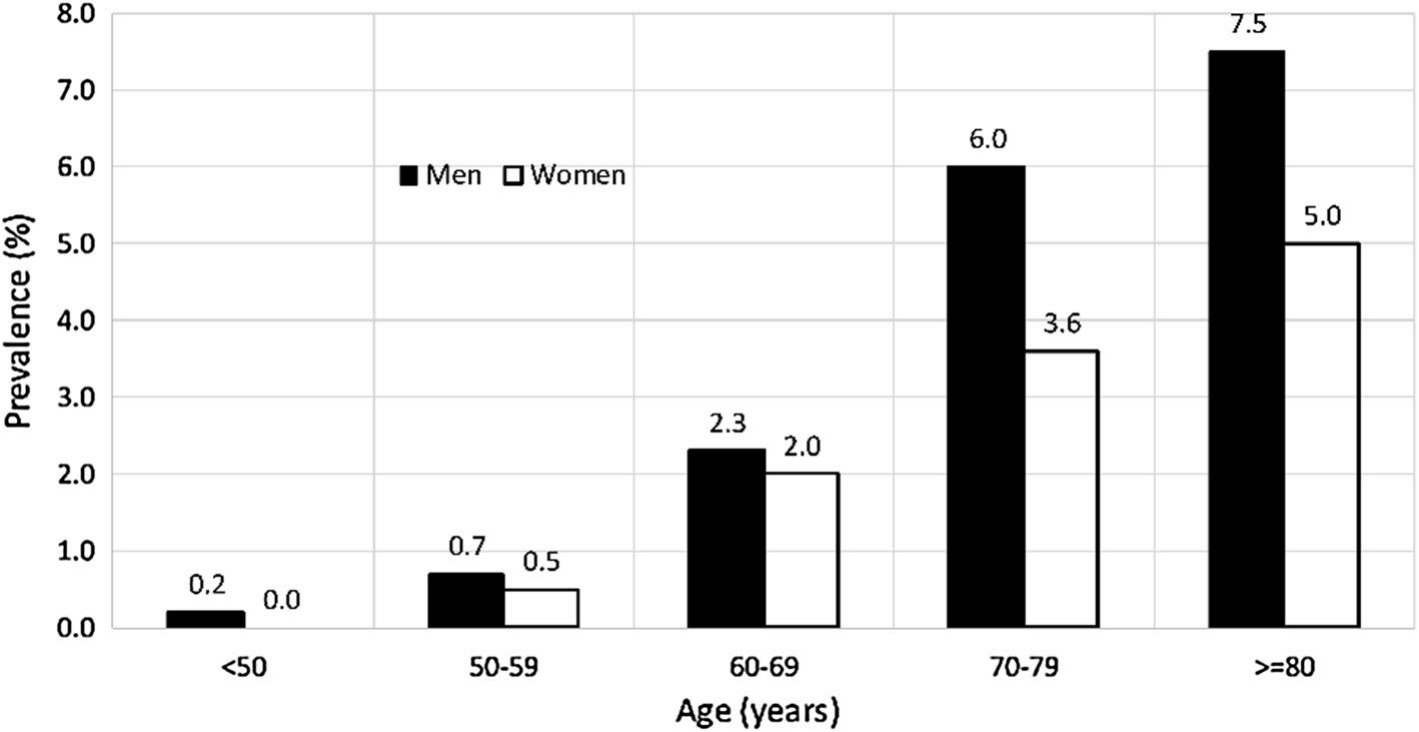
Prevalence of moderate carotid stenosis cases among men and women in various age groups [5]

Cardiovascular diseases (CVDs) are the medical conditions affecting heart, blood or blood vessels. In clinical medicine, coronary artery disease, carotid artery disease and peripheral vascular disease are common manifestations of CVDs. Carotid artery stenosis (CAS), where the atherosclerotic plaque is formed because of response to physical or metabolic injury, may cause debilitating stroke; hence, its early detection, prevention and treatment are important. According to American Heart Association (AHA), in 2013, stroke was the second leading cause of deaths (6.5 million) globally [1]. In the United States alone, stroke consumes 1.7% of national health expenditures, and by 2030, the total annual cost on stroke is expected to increase by 129% [2]. It is estimated that the patients (total of 1840 patients) with 60% or more stenosis have 16% risk of experiencing a stroke over a period of 5 years [3]. While calculating the age- and sex-standardized incidence rates of strokes (45–84 years age group) from 11 studies (from 1984 to 1995) in 10 different cities of Europe, Russia, Australasia, and the United States, it was found out that there were higher and lower incidence rates of 627 and 638 per 100,000 people in Russia and France, respectively, while a similar incidence rate in the range of 300–500 per 100,000 people was found for the rest of the regions [4]. The prevalence of moderate/severe CAS increases with age (total participants: 23,706), especially after the age of 50, and affecting men more than women [5] (Fig. 1). In a clinical setting, if physician detects the presence of carotid bruit, an ultrasound examination or CTA/MRA is then prescribed to confirm the presence and severity of carotid stenosis. This paper provides a review on the technical development in the imaging modalities for CAS. The article is organized systematically to first discuss the genesis of the CAS, followed by a brief overview on the development of imaging modalities, viz. CTA, MRA, and DUS, their comparative analysis, and prospects. A brief note on the development of potential imaging modalities is also included.

### Carotid artery stenosis (CAS): genesis

**Fig. 2:**
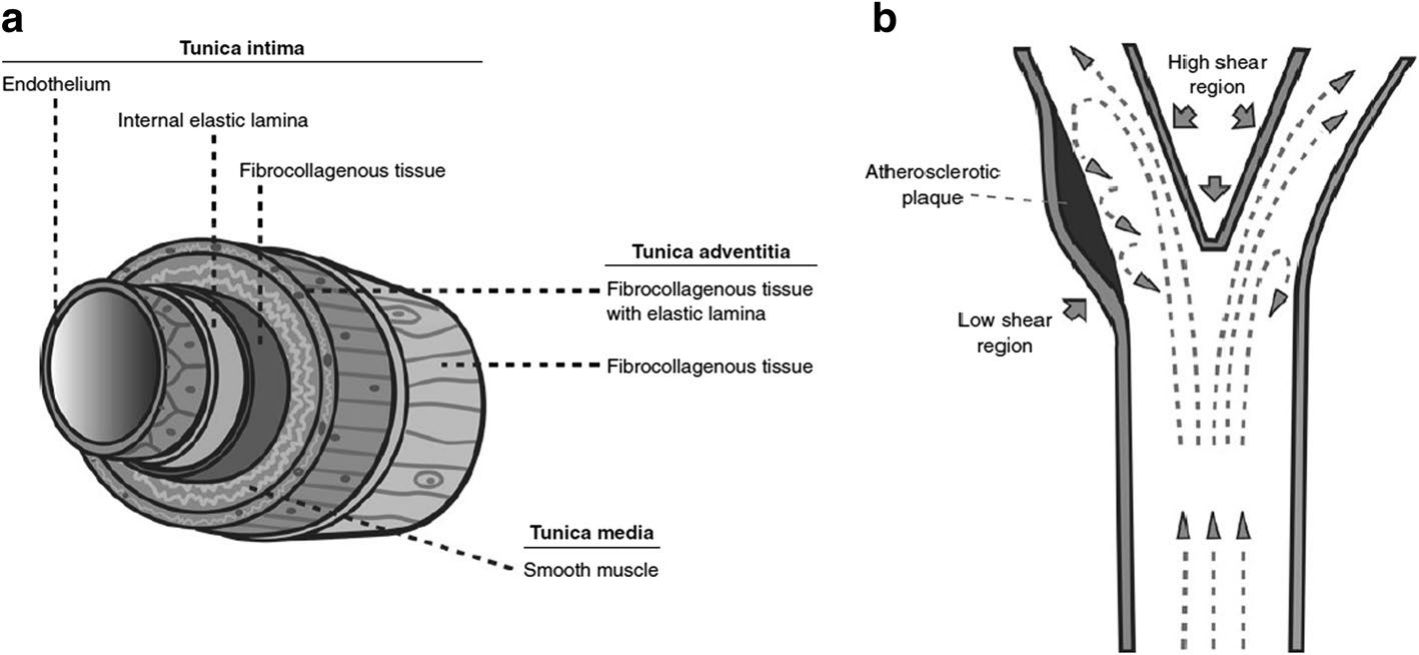
Schematic of **a** blood vessel structure and its components, **b** localized shear stress in carotid artery flow [7]

The structure of an artery, or in most blood vessels like carotid artery, is composed of three layers of tissue, namely, tunica intima, tunica media, and tunica adventitia [6]. As shown in Fig. 2, these layers encase over an open space known as the lumen that holds and directs the blood stream through its designated route. Directly adjacent to the blood flow, the tunica intima contains a monolayer of endothelial cells that forms a sleek surface, facilitating the movement of blood with minimal friction. Surface receptors are embedded into these cells, regulating vascular permeability, platelet aggregation and resistance to thrombosis. In response to a physical or metabolic injury, the endothelium triggers a biochemical signal for inflammatory cell migration to the damage site. Recurring damages in this region result in formation of fatty streaks, leading subsequently to the formation of an atherosclerotic plaque [3]. Lesions inflicted on the arterial wall could be attributed to several factors: hemodynamic, metabolic, environmental, and genetic risk factors, which prompt an inflammatory response through cell proliferation and an eventual plaque progression. The perpetuation of immune response within the arterial wall leads to formation of an atheroma that protrudes into the arterial lumen causing a reduction in luminal diameter. Further cell repair attempts by the endothelial cells, forming the atherosclerotic plaque, constrict the arterial diameter, progressing into an eventual arterial stenosis [7].

Atherosclerotic plaque formation at the carotid bifurcation can be greatly attributed to hemodynamic forces. In a laminar flow of the bloodstream, a gradient of fluid velocities appears increasingly from the wall to the centre. Friction induced between the blood flow and wall creates a tangential force on the wall, which is termed as wall shear stress. With the differing velocities from the geometry of the artery, regions of high and low wall shear stresses are formed (Fig. 2). In the case of the carotid bifurcation, flow separation occurs when high blood velocities meet the divider, giving rise to secondary flow patterns along the external side of the carotid sinus. Through this separation, high shear stress regions are localized near the inner walls of the bifurcation, with low shear stress regions located nearer to the outer walls. The increase of wall stresses promotes cell elongation and alignment with respect to the flow, while exposure to lower stresses induces rounding of these cells. These surface irregularities in the low stress regions of the endothelial layer results in the reduction in cell reparability and hence, promoting plaque formation. In addition, pulsating nature of the blood flow from the cardiac cycle induces varying shear rate, affecting areas with slower flow such as the carotid sinus and narrowed arteries. These low shear rate areas are prone to atheroma formation [7]. Progressive narrowing of the carotid artery or sudden plaque rupture causing thrombus occlusion or embolization may result in transient ischaemic attack (TIA) or stroke.

### X-ray imaging

X-ray imaging dates back to 1895 when Rontgen discovered X-rays for imaging the biological tissues. The intensity of the radiation changes due to scattering and attenuation while transmitting through various tissues (different optical properties). This forms a 2D image when falling on a photographic film. The first medical application was in the field of bone (high attenuation for X-ray photons) imaging [8]. To visualize the blood flow in the arteries, X-ray imaging requires injection of a contrast agent. Contrast agents, like Iodine, Lanthanide, Gold nanoparticle, etc. [9], help in attenuating the X-rays which are measured as contrast density value in Hounsfield units (Hu). This is called X-ray angiography.

### Digital subtraction angiography (DSA)

**Fig. 3:**
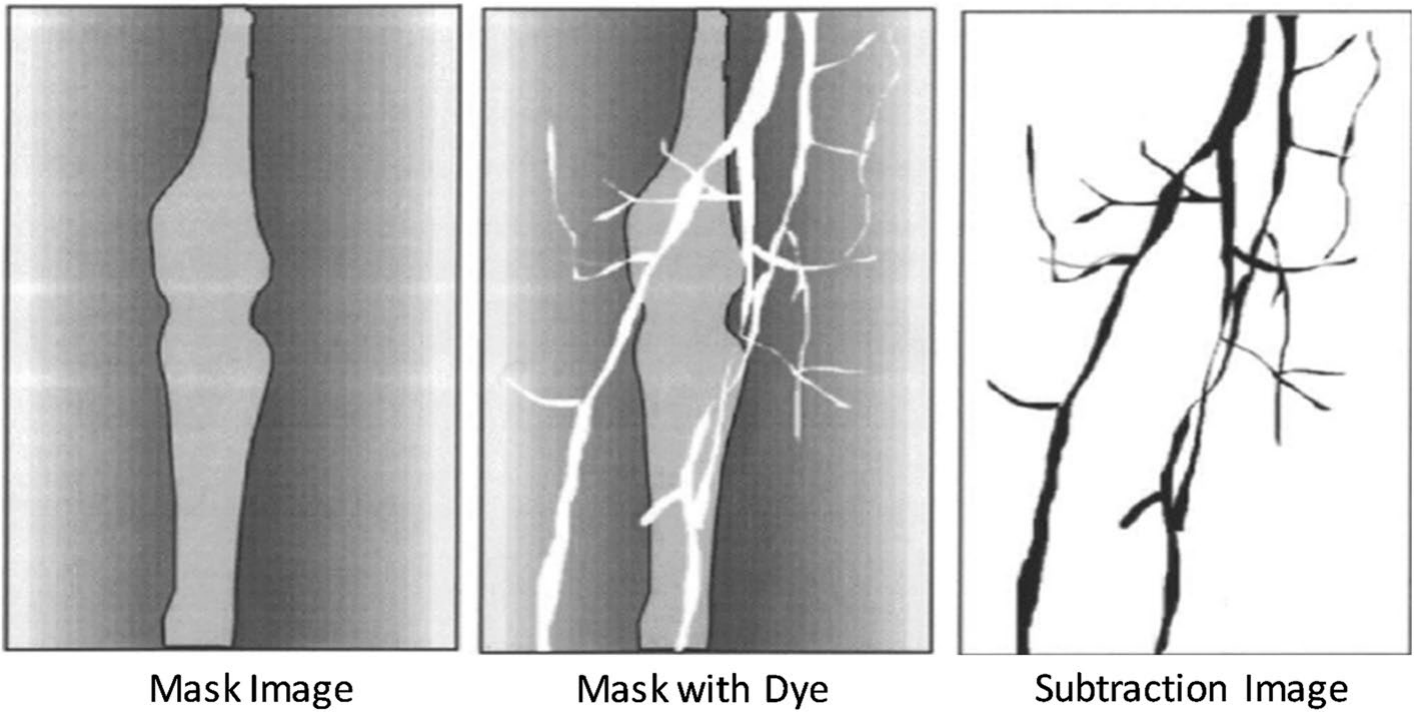
Subtraction image methodology using mask and dye images in DSA [12]

DSA or mask mode subtraction is a technique to specifically visualize the vascular system (blood flow in the vessels) with injection of smaller-than-usual quantity of contrast material (compared to conventional quantity) under an X-ray exposure [10]. In DSA, on subtracting the two X-ray images taken before (mask) and after the injection of contrast material (Fig. 3), a blood vessel image with high contrast is obtained. Comparing the visualization of carotid artery with the conventional angiography (in 100 patients), DSA provides a sensitivity, specificity, and accuracy of 95%, 99%, and 97%, respectively [11].

**Fig. 4:**
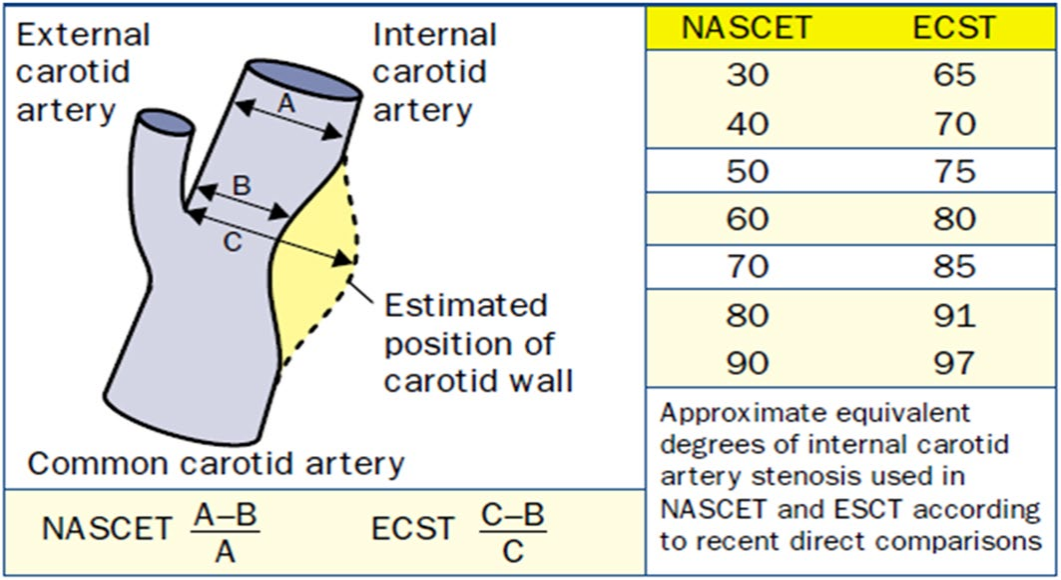
NASCET and ECST measurements of internal carotid artery stenosis [18]

DSA was used in the randomized studies in the European Carotid Surgery Trial (ECST) [13] and North American Symptomatic Endarterectomy Trial (NASET) [14, 15], comparing the surgical carotid endarterectomy (CEA) versus medical therapy in the treatment of patients with moderate (50% to 69%) and severe stenosis (70% to 99%), as defined using the NASCET criteria. Some confusion was introduced to the field because of the different criteria in defining the degree of stenosis. NASCET criteria of 50% stenosis is roughly equal to 75% stenosis by ECST criteria (Fig. 4). However, both studies found that compared with medical therapy, there was significant benefit with CEA in symptomatic patients with severe degree of stenosis (NASCET 70–99% stenosis, and ECST 80–99% stenosis). There was only modest benefit in those with moderate degree of carotid stenosis (NASCET 50–69% stenosis, ECST 70–79% stenosis). DSA is also useful in predicting the cerebral hyperperfusion phenomenon (HPP) developed upon carotid interventions. HPP is of low incidence but it can lead to morbidity and mortality, and may cause watershed cerebral infarction [16]. Indirectly measuring the cerebral circulation time (CCT), using the time to reach maximum contrast intensity, DSA can predict HPP (prolonged CCT) with a sensitivity and specificity of 75% and 100%, respectively [17].

### Computed tomography angiography (CTA)

**Fig. 5:**
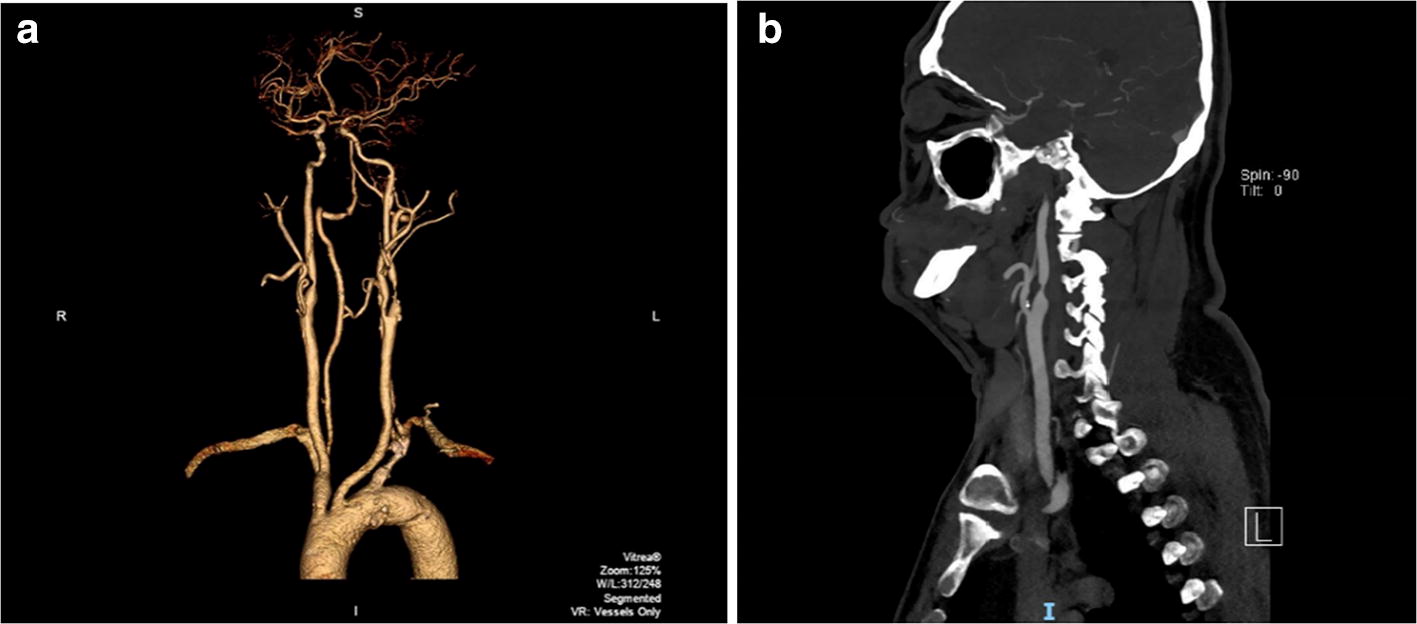
Carotid artery CTA: **a** 3D reconstruction, **b** 2D slice image (credits: National Heart Center Singapore)

Since the conventional angiography involves acquisition of a 2D image from a 3D body, it superimposes imprints of all the structures across the 3D body as the X-rays pass through it, and therefore, a lot of information is not completely revealed. To address this issue, in 1973, the first successful CT scanner, claimed to be 100 times more sensitive compared to conventional X-ray angiography, was introduced [19]. In CTA, slice-based imaging of the 3D body is done, wherein a series of 2D slice (of defined thickness) images are generated for the whole 3D body. From the 2D slices, a 3D reconstruction can be performed for a better visualization (Fig. 5). The equipment consists of an X-ray source that irradiates a narrow section of the 3D body (called slice) from one side, while the transmitted signals are received by the detector on the other. To perform a complete 360° scan, the combined set of source and detector rotates around the body to produce slice by slice images. This basic technique to perform CTA has gone through a series of developments in terms of scanning speed, slice-to-volume scanning, cone-beam scanning, etc. as chronologically summarized in the work of Kalender [20]. Revolutionizing the medical imaging, CTA has further reduced the quantity of contrast agent needed for the examination compared to the conventional angiography [21].

**Fig. 6:**
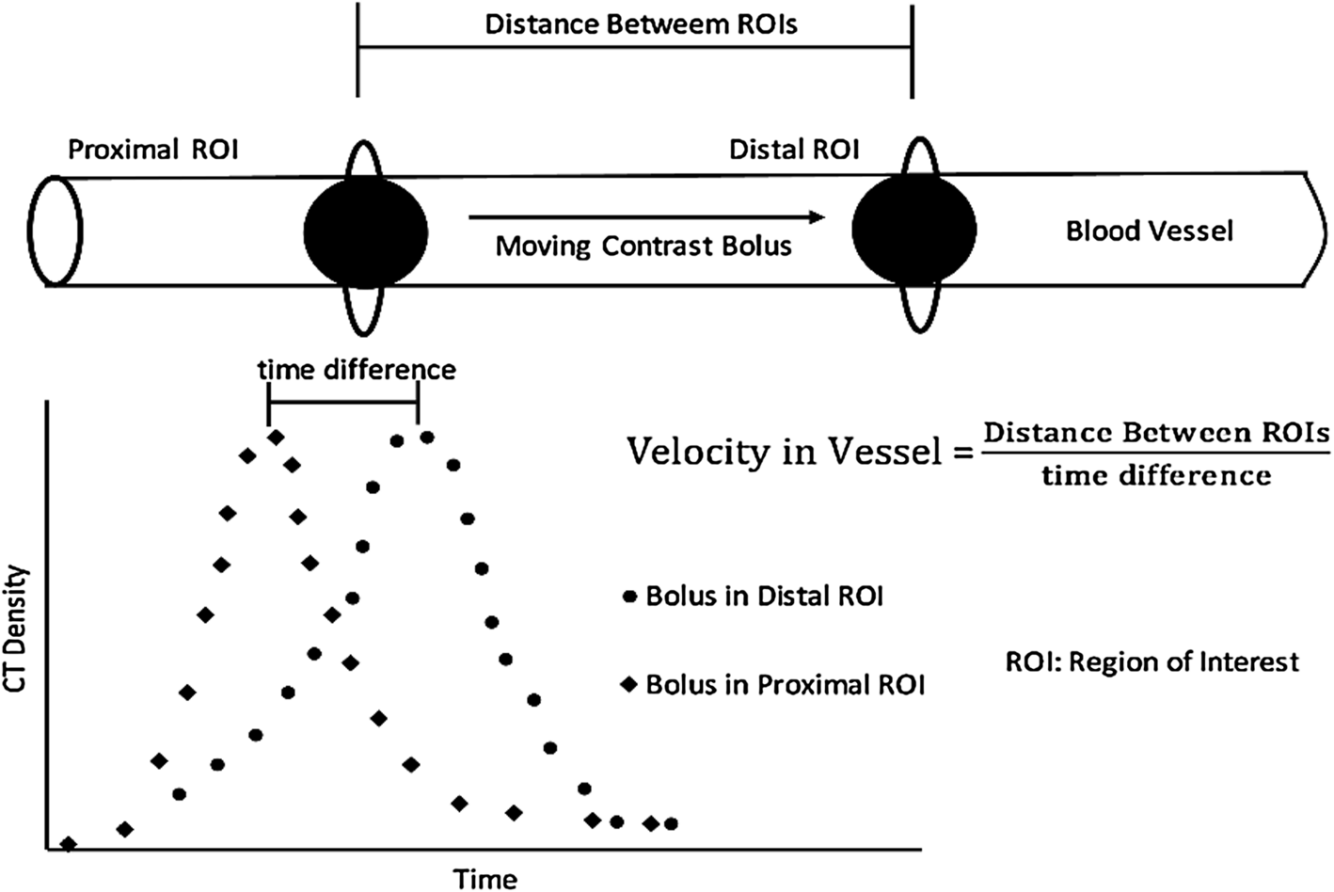
Contrast bolus intensity-based time-of-flight method to estimate velocity with two distal region of interests (ROIs) [28]

With the advancement in the image processing and analysis techniques, semi- and fully automated 3D CTA analysis programs were developed and tested [22]. In the early attempts, where a semi-automated image processing algorithm is used to segment and trace the target blood vessel, CTA performed poorly compared to DSA [23] or duplex ultrasound [24]. In semi-automated algorithms, manual identification of the reference point of segmentation and appropriate selection of upper and lower bound of contrast intensity (in Hu) are the necessary steps. Further, with the post-processing manual corrections, semi-automated CTA showed an improvement of 55% in the correlation coefficient (*r*: 0.53 to 0.82) with respect to DSA [25]. Recently, an operator independent fully-automated CTA showed a carotid artery detection rate of 75% on a small dataset of 14 patients [26]. In this method, upon normalizing the original slice images, an automatic identification of the circular region of interest, in the threshold and diameter range of 200–450 Hu and 2–6 mm, respectively, which corresponds to the carotid artery marking was performed. Through an inverse approach, where the bone region is first segmented and eliminated before segmenting out the carotid artery, using machine learning-based *k*-NN (*k* nearest neighbour) classifier model, a full-automated CTA showed an accuracy of 99% [27]. Moving on, it is believed that velocity evaluation can further strengthen the diagnosis efficacy of CTA. The quantification of the visualized blood vessels in terms of blood flow velocity measurement can be done with the help of complex algorithms, viz. tracking and computational methods. These algorithms track the progression of contrast material with respect to time (Fig. 6) [28]. While tracking method tracks a contrast material as it moves inside the vessel within the field of view, computational method employs the mass conservational laws to compute the flow rate.

### Magnetic resonance imaging (MRI)

Magnetic resonance imaging (MRI), where radio frequency pulses are used to stimulate the proton spinning in a tissue (with varied material properties with respect to the surrounding), is most widely used to achieve high contrast images of the body’s internal structures [29]. In principle, when spinning protons experience an external constant magnetic field, they align themselves in the same direction of the applied field, while few protons, possessing high energy state, will align in the direction opposite to applied magnetic field. This gives a resultant magnetization in the longitudinal direction (align with external magnetic field). Further, on the enforcement of a 90° radiofrequency energy pulse at resonance frequency, the protons aligned with external magnetic field absorb the excess energy and change their alignment directions to high energy state. In such a scenario, the spinning of the protons will be in synchronization with each other, which will give a resultant magnetization in the transverse direction to the external magnetic field. On the removal of the radiofrequency signal, the protons will fall back to their original state and releases energy, in the form of magnetic resonance signal, at a rate determined by either *T*_1_ or *T*_2_ relaxation times [30]. As the radiofrequency signal is removed, the spinning protons will start repelling each other, which leads to the decay of net transverse magnetization achieved earlier. This decay processes is measured in terms of *T*_2_ relaxation time (or spin–spin relaxation). On the other hand, the protons in the higher energy state will start falling back to the lower energy state in the direction of external magnetic field, which will lead to the re-growth of the resultant magnetization in the longitudinal direction, which is measured in terms of *T*_1_ relaxation time (spin–lattice relaxation). Based on the molecular structure under study, different *T*_1_ or *T*_2_ times are evaluated, and hence forms the basis for forming an image. This image provides a comprehensive information on internal structures and boundaries.

### Magnetic resonance angiography (MRA)

In addition to basic MRI technique, registration of flow information led to the development of Magnetic resonance angiography (MRA). To bring the image contrast pertaining to the blood movement in the vessels and suppress the static tissue around, MRA projection imaging is used. Methods like temporal subtraction, inversion excitation, stimulating the adjacent regions, and phase shift are used create projection images [31]. In temporal subtraction, two images are taken and subtracted with a similar static tissue signal and dissimilar moving tissue (blood) signal to produce a contrast-enhanced moving tissue image. For instance, in phase-contrast (PC) method [32], two images, at the systolic and the diastolic instants of the velocity waveform, are used to create a high contrast image. While the fast-moving blood during systolic instant leads to a signal loss, there will be a relative retaining of the signal for diastolic instant image. On the other hand, signal from static nearby tissue remains consistent. Hence, subtracting the two images results in a bright image of the blood vessel that helps in locating the lesion. Another method is time of flight (TOF) [32] where selective inversion magnetization is applied to the spin of the flowing tissue molecules and the two images with and without inversion are subtracted to leave out the static tissue signal. Alternative to the temporal subtraction method, a similar cancellation technique has also been developed, which induces the suppression of the static tissue and selectively exciting the moving tissue to produce the desired projections [33].

Both PC and TOF methods are widely used for the detection of carotid artery diseases. Using these methods, under both NASCET and ECST criteria, the percentage of stenosis detection (in 55 patients) is found to be higher than DSA [34]. In a study [35], comparing the sensitivity and specificity of TOF, high-resolution (HR) contrast-enhanced (CE), and time-resolved CE MRA techniques in 21 patients, it was found out that the sensitivity for all the techniques was 100%, while specificity was the highest for TOF MRA (96.7%). Meta-analysis has shown that the overall sensitivity and specificity of TOF MRA to diagnose severe stenosis (70% ≤ *S* ≤ 99%) are 91.2% and 82.3%, respectively [36]. Contrast agents like gadolinium is also used to produce better-quality images for smaller vessels which are likely to be not visible with normal MRA [37]. Further, use of automated vascular segmentation computer-aided programs helps in determining the accurate dimensions of the vessels [38, 39]. In an in vitro stenotic phantom-based study, using a waveprop segmentation method, wherein the algorithm calculates the propagation of a virtual wave to segment the vessel upon manual identification of the start and end points of a 3D path line, the semi-automated MRA showed a high correlation coefficient (*r*) of 0.99 with the true diameter [40]. In an in vivo study (40 carotid arteries), using another semi-automatic MRA, wherein a user-defined volume of interest and a point within the vessel is needed to extract the vessel central axis followed by the detection of the vessel boundaries, a varied degree of agreement with respect to the radiologist’s visual inspection was found [41]. While arteries with 0–49% stenosis were detected with 100% agreement, arteries with 50–69% and 70–99% stenosis showed agreement of 57% and 77%, respectively. In a recent first of its kind attempt, using a hierarchical-tree model along with the application of *k*-NN supervised classifier to detect the lumen boundary, in both normal (15) and atherosclerotic (20) subjects, a similarity index (Dice overlap), between the software and the manual delineation, of 0.87 was achieved [42].

### Duplex ultrasound

Being an accurate, noninvasive, yet low-cost mode of diagnostic imaging, duplex ultrasound (DUS) has become one of the main assessing methods for carotid diseases. This technique essentially fuses two procedures—traditional B-mode (greyscale) ultrasound, wherein images of the body parts/tissue architect, at rest, are generated from the reflected sound waves; and colour-Doppler ultrasound that visualizes the motion of the moving parts or fluid (blood) to measure the speed and other flow parameters. B-mode or brightness mode ultrasound is basically a two-dimensional (2D) cross-sectional image, generated by the reflected ultrasound waves (echo) from the tissue structure boundaries, upon transmission of short burst or pulse of ultrasound by the transducer placed on the outer surface of the body. This is called pulse–echo principle. The so-formed 2D image is composed of several B-mode lines that arises from a respective pulse–echo sequence. A pulse–echo sequence is formed by the display of the brightness spot generated by the target that reflects the pulse, located at further depths from the transducer [43].

**Fig. 7:**
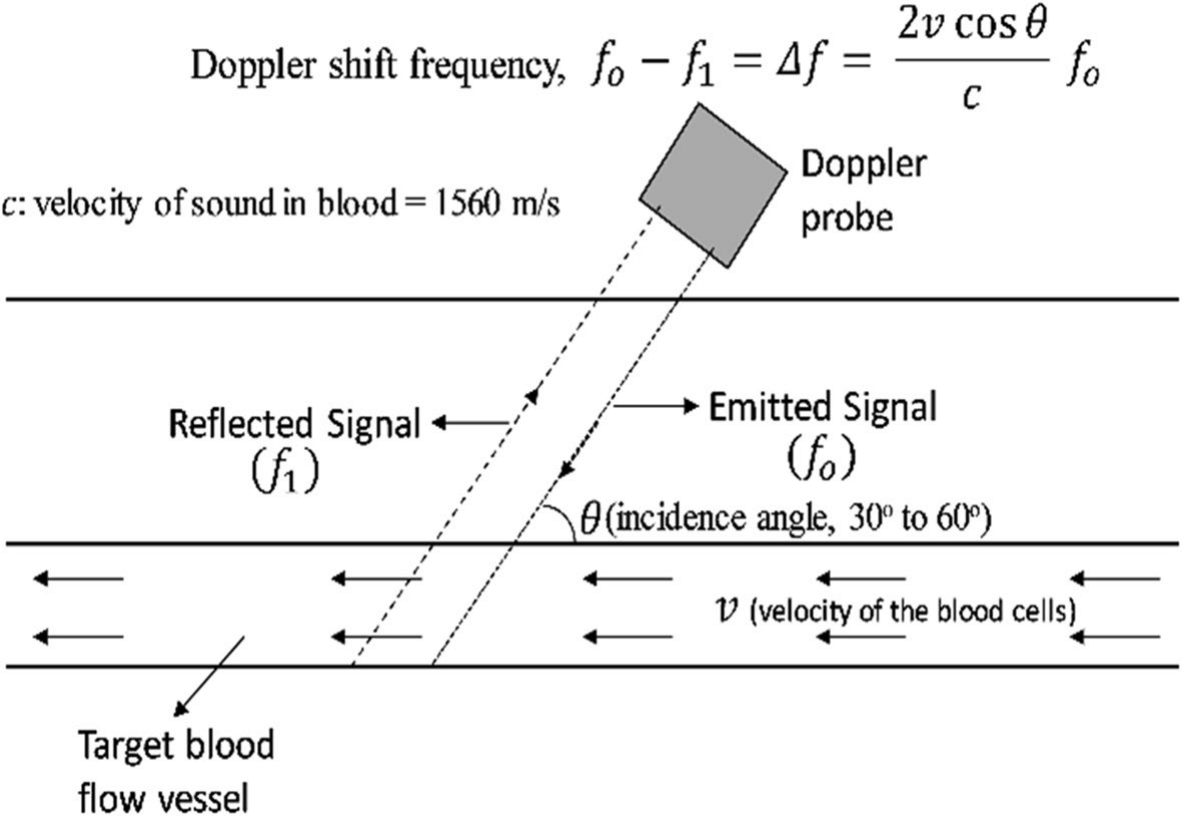
Schematic of Doppler ultrasound principle

**Fig. 8:**
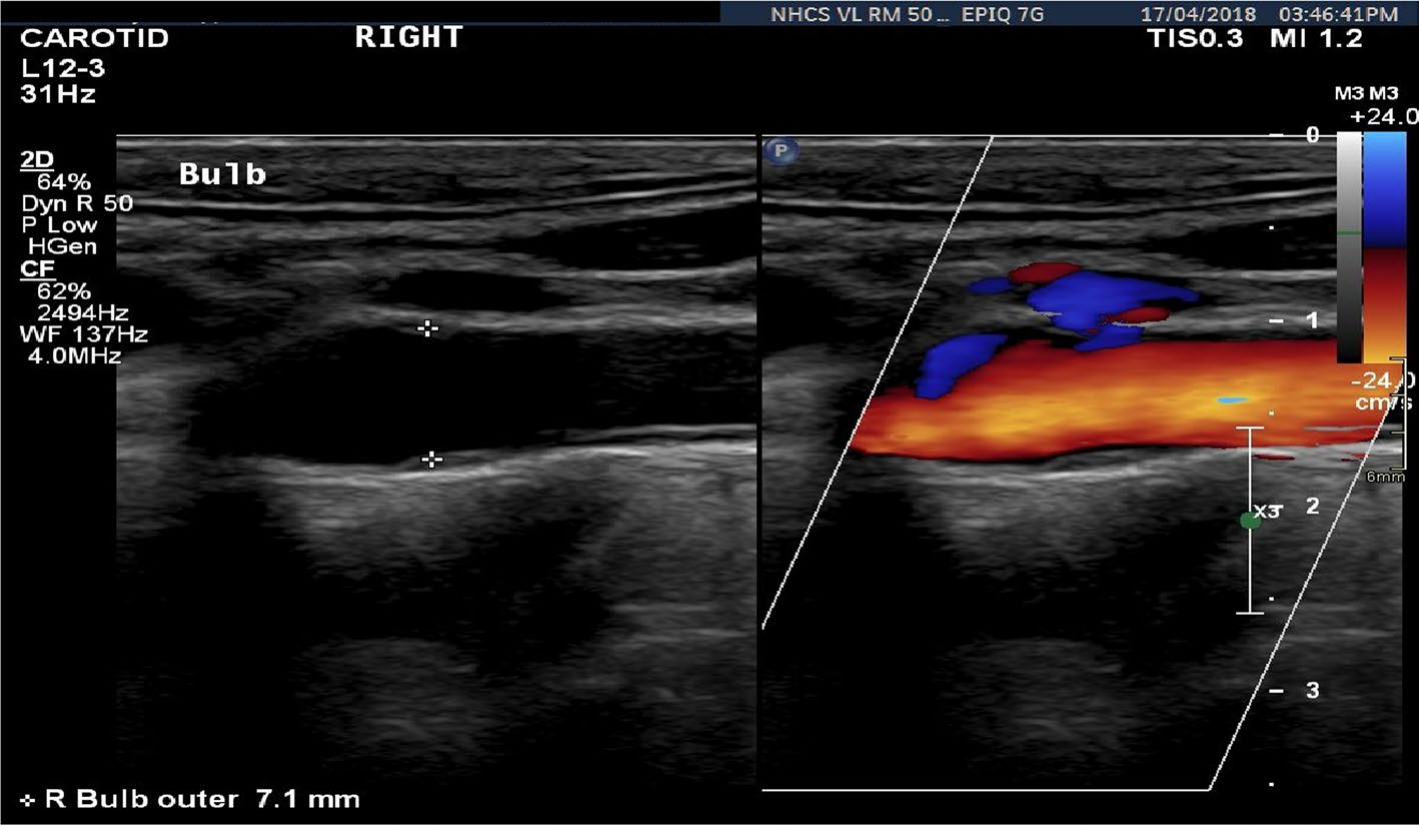
A duplex ultrasound output displaying the B-mode and colour flow doppler image side-by-side (credits: National Heart Center Singapore)

First reported by the Austrian physicist, Christian Doppler, Doppler effect (DE) or Doppler shift (DS) found numerous applications in the field of astronomy, radar, flow measurement, satellite communication, etc. Utilizing the basic principle of frequency shift in the sound waves due to relative motion of either the observer or the source of sound travelling in a medium, DS in ultrasound was first discovered in 1957 (Fig. 7). Based on operation and functionality, there are broadly two methods of Doppler ultrasound, namely, continuous-wave Doppler (CWD) and pulsed-wave Doppler (PWD). As the name suggests, in CWD, a continuous ultrasound wave is emitted and received to scan the whole moving target area, while PWD involves sending short pulse of ultrasound using frequency range gating method [44], and processes a small area for velocities at a specific field depth. CWD results in tracking high velocities but at the expense of loss of spatial velocity information, and vice versa for PWD. The greyscale B-mode image, superimposed with blood flow information, is displayed on the same screen to facilitate a more realistic assessment of the internal anatomy (Fig. 8). The presence of a stenosis consequently appears in the greyscale images with the increasing blood velocity, thereby forming a basis/criterion for the noninvasive stenosis evaluation [45]. Characterizing the blood flow in the artery, Doppler ultrasound plays an important role in improving the CAS diagnosis [46, 47]. Additional colour flow imaging in conjugation with Doppler imaging has revolutionized the visualization of the blood flow velocities in the arteries and veins [48]. In a retrospective study on 1390 patients, DUS has proven to provide a fast screening and surgery decision making within 2 weeks of hospitalization [49].

Changes in the diameter of the carotid artery, because of plaque formation, is visualized in the B-mode images to diagnose CAS. Intima-media thickness or IMT measurement by B-mode ultrasound, which closely relates to those obtained from the histological results, was the first breakthrough by Pignoli et al. [50] in 1986. Since then, based on B-mode ultrasound IMT evaluation, many studies have been performed to study the correlation between the occurrences of carotid atherosclerosis versus age, sex, race, diabetes, pulse rate and cigarette smoking [51]. Since B-mode diagnosis is dependent on the IMT [52], an accurate marking of the wall boundaries is a critical factor in the diagnosis. To minimize inter- and intra-observer variability in manual IMT identification (by 2–4 times), an automated computerized edge detection algorithm, where maximum intensity gradient point in the perpendicular direction of the manual path created along the vessel boundary, was introduced by Selzer et al. [53] in 1994. Thereafter, many such algorithms, like dynamic programming procedure, model-based algorithm, match filter algorithm, etc., were developed to more accurately estimate IMT [54]. Using a completely user-independent deep learning-based highly repeatable and reproducible arterial layer identification method to segment the IMT, a linear regression between the estimated and ground truth values gives $R^{2}$ a value of 0.98 [55]. For an obvious reason, this method is, however, computationally expensive. On the contrary, in a recent study, using a state-space equation-based algorithm, a robust IMT segmentation model gives $R^{2}$ a value of 0.92 [56]. Therefore, a trade-off between the computational cost and accuracy is inevitable.

**Fig. 9:**
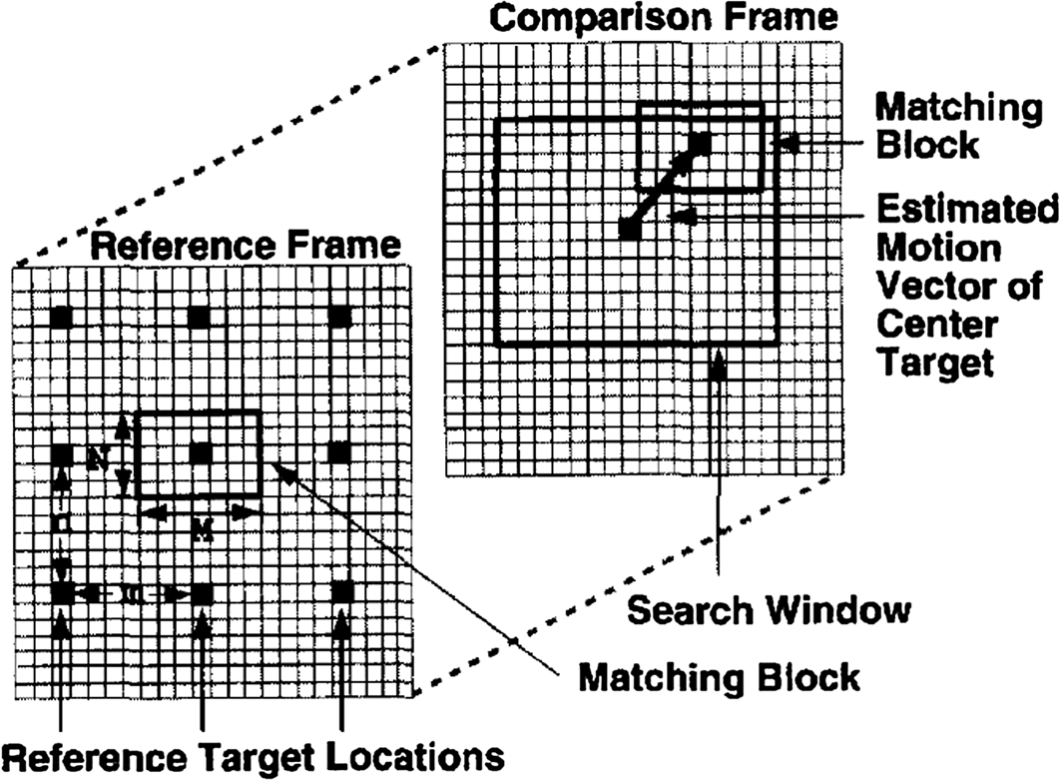
Schematic on block-matching (BM) methodology [57]

Another breakthrough in the diagnosis of the atherosclerosis diseases is the evaluation of the degree of stiffness of the arterial wall as a measure of pathologic condition. Measuring this stiffness factor on 60 left and right post-mortem carotid arteries, ultrasonic phase-locked echo-tracking system, with a correlation factor of 0.68, correlated the stiffness factor and the extent of atherosclerosis [58]. This leads to the development of algorithms, like Block Matching (BM), to perform auto-IMT segmentation and arterial wall stiffness analysis. As shown in Fig. 9, BM is performed by selecting a block of pixels or kernel in the current image (reference block) and a similar block (most similar kernel block in terms of intensity [59] that is assumed to be constant over time) is identified in the successive frame [60]. Displacement between these two successive blocks is evaluated to track the motion of the reference block. Since it is expected that the intensity of the reference block (or appearance of the target) will change over time, an Adaptive BM (ABM) method has evolved, whereby, to accurately perform the motion analysis, the reference block is replaced consecutively as the algorithm processes the successive images [61]. Recently, to compute the 2D motion of the carotid wall, BM was applied in conjugation with a state-space equation derived from a linear elastic model of the carotid artery wall [62]. While doing so, a correlation coefficient of 0.98 and 0.95 for the radial and longitudinal motion, respectively, was reported. Further, using a nonlinear state-space equation, an improvement to 0.99 in the correlation coefficient was achieved [63].

### Comparative analysis

**Fig. 10:**
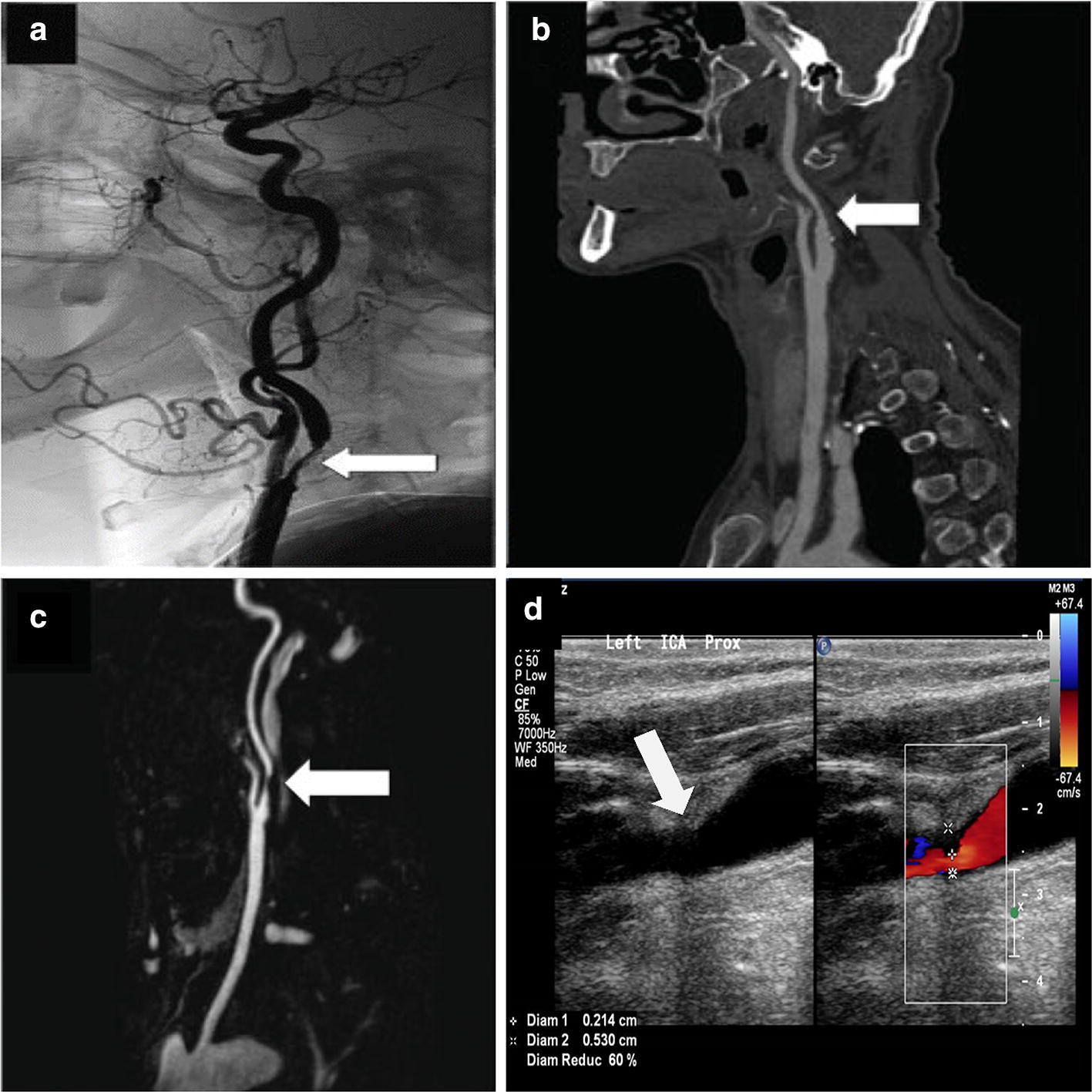
ICA stenosis visualization with a white marker arrow in **a** DSA image [75], **b** CTA image [75], **c** MRA image [75], and **d** DUS image (credits: National Heart Center Singapore)

Despite being considered as the gold standard to diagnose carotid stenosis, the invasive DSA incurs an increased risk of stroke up to 1.2% [64]. Hence, other noninvasive imaging methods are generally used as the first-line screening method. A methodological characteristic comparison between CTA, MRA and DUS is given in Table 1. Figure 10 shows the stenosis visualization in various imaging modalities. While DSA, CTA and MRA provide a full view of the vasculature, DUS only provides a small sectional view of the artery. It can be observed from Table 2 that the sensitivity and specificity of DUS to diagnose > 70% stenosis increases with the increasing sample size and age spectrum of the study population. Given the higher prevalence of CAS in the older age group (> 70 years) [65], the study performed by Huston et al. [66], with a wider range of age group (14 to 88 years), provides a higher specificity of detecting patients with no stenosis by DUS. This is further corroborated by the fact that specificity value decreased by 15% as the age group spectrum is shifted towards older age group (39 to 88 years) in the work by Nederkoorn et al. [67].

**Table 1:** Methodological characteristic-based comparison among the existing imaging modalities [68–71]

Diagnostic test	Methodology	Advantage	Limitations/drawbacks
Computed tomography angiography (CTA)	Use of high-dose X-rays to get a detailed picture of the vascular system and its functioning Patients may also have a dye injected to make it easier to spot blockages	The exam is quick 3D results Able to identify a wide variety of abnormalities	Risk associated with radiation exposure and contrast use (e.g. allergic reaction, contrast-induced nephropathy) Incidental findings may lead to unnecessary further tests Presence of vessel calcification leads to an inaccurate stenosis estimation Large area is needed to house the equipment
Magnetic resonance angiography (MRA)	Make use of large magnets and radio waves to take pictures of internal organs like heart and arteries	Does not involve exposure to ionizing radiation	Not safe for patients with implants that are not MRI-conditional Time consuming Not suitable for patient with claustrophobia Not widely available Contrast associated adverse reaction (e.g. nephrogenic systemic fibrosis in patients with kidney impairment)
Duplex ultrasound (DUS)	To check the blood flow to the brain for a probable plaque formation in the carotid artery	Reasonable tool in picking up carotid atherosclerosis	Require trained personnel to perform and interpret Reflect the presence of systemic atherosclerosis

**Table 2:** Sensitivity and specificity comparison between duplex ultrasound, CTA and MRA

Studies	Number of subjects (*N*)	Age group (years)	Severity (*S*)	Sensitivity (%)	Specificity (%)	Remarks
Computed tomography angiography (CTA)						
Marks et al. [76]	14	49–84	0% ≤ S ≤ 30%	86	–	CTA results were 89% accurate compared to conventional angiography
			30% ≤ S ≤ 69%	86		
			70% ≤ S ≤ 99%	100		
Farres et al. [77]	24	48–88	50% ≤ S ≤ 99%	100	95.2	Sensitivity—95% CI, 15.8% to 100% Specificity—95% CI, 83.8% to 99.4%
Anderson et al. [78]	40	44–83	50% ≤ S ≤ 99%	89	91	For mild stenosis (0–29%) and occlusion, CTA was found to be almost 100% accurate
Koelemay et al. [79]	864 (meta-analysis)	55–73	70% ≤ S ≤ 99%	85	93	Sensitivity—95% CI, 95% CI, 79% to 89% Specificity—95% CI, 89% to 96%
Wardlaw et al. [74]	372 (meta-analysis)	–	70% ≤ S ≤ 99%	77	95	Sensitivity—95% CI, 68% to 84% Specificity—95% CI, 91% to 97%
Magnetic resonance angiography (MRA)						
Cosottini et al. [80]	92	45–82	0% ≤ S ≤ 99%	97	82	The patients were clinically and ultrasonically tested for stenosis sign earlier
Nederkoorn et al. [67]	350	39–88	70% ≤ S ≤ 99%	92.2	75.7	Sensitivity—95% CI, 86.2% to 96.2% Specificity—95% CI, 68.6% to 82.5%
Nederkoorn et al. [73]	Meta-analysis	–	S < 70% versus 70% ≤ S ≤ 99%	95	90	Pooled weighted analysis Sensitivity—95% CI, 92% to 97% Specificity—95% CI, 86% to 93%
Wardlaw et al. [74]	380 (contrast enhanced MRA)	Meta-analysis	70% ≤ S ≤ 99%	94	93	Sensitivity—95% CI, 88% to 97% Specificity—95% CI, 89% to 96%
	774 (MRA)			88	84	Sensitivity—95% CI, 82% to 92% Specificity—95% CI, 76% to 97%
Duplex ultrasound (DUS)						
Huston et al. [66]	621	14–88	50% ≤ S ≤ 70%	86.4	90.1	Peak systolic and end diastolic velocity of 230 cm/s and 70 cm/s, respectively, were evaluated for stenosis ≥ 70%
			70% ≤ S ≤ 99%	92.1	89.5	
Nederkoorn et al. [67]	350	39–88	70% ≤ S ≤ 99%	87.5	75.7	Sensitivity—95% CI, 82.1% to 92.9% Specificity—95% CI, 69.3% to 82.2%
Nederkoorn et al. [73]	Meta-analysis	–	S < 70% versus 70% ≤ S ≤ 99%	86	87	Pooled weighted analysis Sensitivity—95% CI, 84% to 89% Specificity—95% CI, 84% to 90%
Jahromi et al. [81]	Meta-analysis	–	50% ≤ S < 70%	98	88	Standards for Reporting of Diagnostic Accuracy (STARD) criteria was used for the study
			S ≥ 70%	90	94	
Wardlaw et al. [74]	916 (meta-analysis)	–	70% ≤ S ≤ 99%	89	84	Sensitivity—95% CI, 85% to 92% Specificity—95% CI, 77% to 89%

CTA and MRA are found to be equally sensitive but differ in specificity—with the specificity of CTA being always higher (Table 2). This could be because of small sample size, for a similar age group, in case of CTA (*N* = 24 to 40) compared to MRA (*N* = 92 to 350). Moreover, MRA suffers from the flow signal loss, mainly in case of turbulent flow and slow flow, leading to an inaccurate measurement of stenosis degree [72]. In such a scenario, contrast enhanced MRA has an advantage. Compared to conventional MRA, contrast enhanced MRA was found to have 7% and 11% higher sensitivity and specificity in diagnosing carotid artery stenosis of 70% or above [73]. Meta-analysis studies of all the three imaging modalities show that MRA is the most sensitive diagnosis tool for carotid stenosis, followed by DUS, with CTA being the least sensitive. In terms of specificity, both MRA and CTA are reasonably good (in the range of 90% to 95%), followed by DUS [74]. CTA has radiation exposure compared to DUS or MRA. Accounting for the patient convenience, operator’s skill requirement, availability, and examination cost, DUS is considered to be the preferred choice for initial screening of CAS.

### Current trends and prospects

Other than radiation exposure, calcification artefact is another technological drawback with CTA. For this, a computer-aided program that potentially addresses the calcification artefacts issue by selectively removing the bone pixels from the CTA image can be used [82]. To measure the plaque volume and its association with ulceration, a multi-detector CTA (MDCTA), which comes with a modest interobserver variability, has been evolved [83]. While detection of vulnerable carotid plaque remains an elusive goal and accurate measurement of blood velocity in CTA is still under development, radiation exposure associated with CTA is always a concern. Other than the degree of stenosis, plaque characterization (lipid level, fibrous cap, plaque haemorrhage, calcification, etc.) and progression [84] are important considerations in clinical management decisions. With high sensitivity and specificity, MRA may better characterize the vulnerable plaque due to its apparent ability to produce high contrast images of the soft tissues [85]. However, with the clinically applicable 1.5-T MRI machine, differentiation among various intraplaque tissues is still a challenge due to low signal-to-noise ratio. Higher field magnet (3-T or 7-T) could possibly improve the signal-to-noise ratio [86]. In a recent study, intraplaque haemorrhage volume, using a 3-T field strength MRI, was strongly correlated to the histology examination (Cohen’s kappa value of 0.76) [39]. This will, however, further increase the cost of the equipment; limiting wide clinical adoption. Imaging the arterial wall at molecular level with the ultra-small superparamagnetic iron oxide particles (USPIO) is a potential method of characterizing the vulnerable plaque through MRI [87]. In such an attempt, a signal loss in MRI due to accumulation of USPIO at the lesion site (high macrophage content), which can be correlated to the presence of plaque prone to rupture, was shown in the past [88]. However, there is a major drawback in terms of time of accumulation of USPIO at the lesion site, which could go up to 24 h [89]. As a potential future direction of research, correlation between cerebral damage which can be accurately detected by MRI and presence of high grade carotid stenosis (> 50%) could prove to be an efficient method of CAS diagnosis [90].

Deriving the geometry features from the CTA and blood flow parameters from MRI [91], a patient-specific carotid hemodynamic can be studied using computational fluid dynamics (CFD) [92]. In the past, it is shown that CFD-based flow analysis can facilitate an accurate and early prediction of the plaque prone to rupture [93]. Moreover, utilizing the CTA and MRI data, in vitro investigation of the carotid flow in determining the post-stenting success is another prediction-based application in the clinical field [94]. In the era of multi-modality patient-specific imaging data availability, CFD tool is evolving as an important milestone in the clinical decision making [95, 96]. Evidence of atherogenesis of the vulnerable plaque linked to the CFD-based hemodynamic parameters such as wall shear stress [97] and pressure gradient [98], was explored in the past. In contrast to the computational cost, the use of an accurate clinically measured inlet boundary condition and calculation of an appropriate outlet boundary condition are, however, very important for the accuracy of CFD-based hemodynamic predictions [99]. For instance, compared to the pure resistance-based outlet boundary condition, use of a lump parameter model showed an improvement in the predicted velocity error, with respect to the measured velocity, from 43 to 16% [100].

Though low cost, low risk and widely available, DUS suffers from inter/intra-observer variability [101]. One way to minimize the variability is the use of computer-aided algorithms to estimate the carotid intima-media thickness (CIMT) based on pixel contrast value [102]. Improper insonation angle, low blood flow rate, and deeper artery location [103] further introduces difficulty to DUS examination of CAS. Helpful in overcoming these shortcomings is the Contrast-Enhanced Ultrasound (CEUS), where an intravascular contrast agent consisting of microbubbles (1–8 µm) filled with perfluorinated gas (low solubility) is injected to acquire high contrast ultrasonic images of carotid artery [104]. In one of its first long term clinical studies (2 years), CEUS was tested on 85 patients, wherein, compared to conventional carotid angiography, a 100% sensitivity, specificity, and accuracy was achieved [105]. The contraindication of administering an ultrasound contrast agent includes heart failure, acute coronary disease or acute myocardial infarction, ventricular arrhythmias, and unstable respiration. Measuring the plaque strain during the cardiac pulsation, DUS can be correlated to the measure of physical plaque instability that could potentially be more clinically relevant compared to the measurement of stenosis degree [106]. Either with or without contrast agent, DUS in 2D is highly dependent on the operator’s skills with limited planar information. Therefore, a 3D volume-based plaque measurement could be an effective tool in establishing the reliability of DUS for an accurate CAS diagnosis [107]. A 3D ultrasound provides a detailed volumetric view of the luminal plaque with high repeatability [108]. Compared to 2D ultrasound, it can easily monitor the progression of the plaque ulceration that substantially affects the treatment decision [109, 110]. Moreover, a 3D ultrasound reduces the inter/intra-observer variability to a minimum level [111]. It is expected that an integration of 3D ultrasound with CEUS will further revolutionize the medical imaging.

### Potential imaging modalities

#### Optical coherence tomography (OCT)

In principle, OCT is like an ultrasound. However, light is used to produce an image instead of sound waves. The basic phenomenon lies in low-coherence interferometry as described by Fercher [112] through a mathematical model. Because of the different optical properties (refractive index) of various constituents of the human tissue (water, lipids, protein, etc.), the echo time delay of the reflected light waves (emitted by a super luminescent source) is calculated through interferometry, and an image is produced as a result of reflected waves’ intensity map [113]. Visualization of the microstructures in the arterial wall, the first in vivo imaging of a rabbit aorta, using an intravascular OCT (IVOCT) catheter, showed a reasonable agreement between the imaging results and the histological examination [114]. In one of the very first attempts, OCT was applied during carotid artery stenting on 17 patients, where a successful detection of the fibrous cap disruption, intraluminal thrombus, and plaque protrusion was achieved [115]. Later, a successful IVOCT examination without any neurological or vascular complications on two patients (one male aged 78 years, and another female aged 83 years), was reported. Unlike carotid angiography, IVOCT provides additional information on ulceration, thin-cap fibroatheroma (TCFA), and intraluminal thrombus, which helped in ensuring post angioplasty and stenting success in the patients [116]. Similarly, OCT applied to an 82-year-old male subject, with angiographically proven severe stenosis in the proximal internal carotid artery, showed a lotus root-like appearance at the lesion site, which is formed because of thrombi undergoing recanalization to form lumen [117]. Benefits and safety (due to its invasiveness) of OCT, for CAS diagnosis and intervention guidance, need further study.

#### Photoacoustic tomography (PAT)

**Fig. 11:**
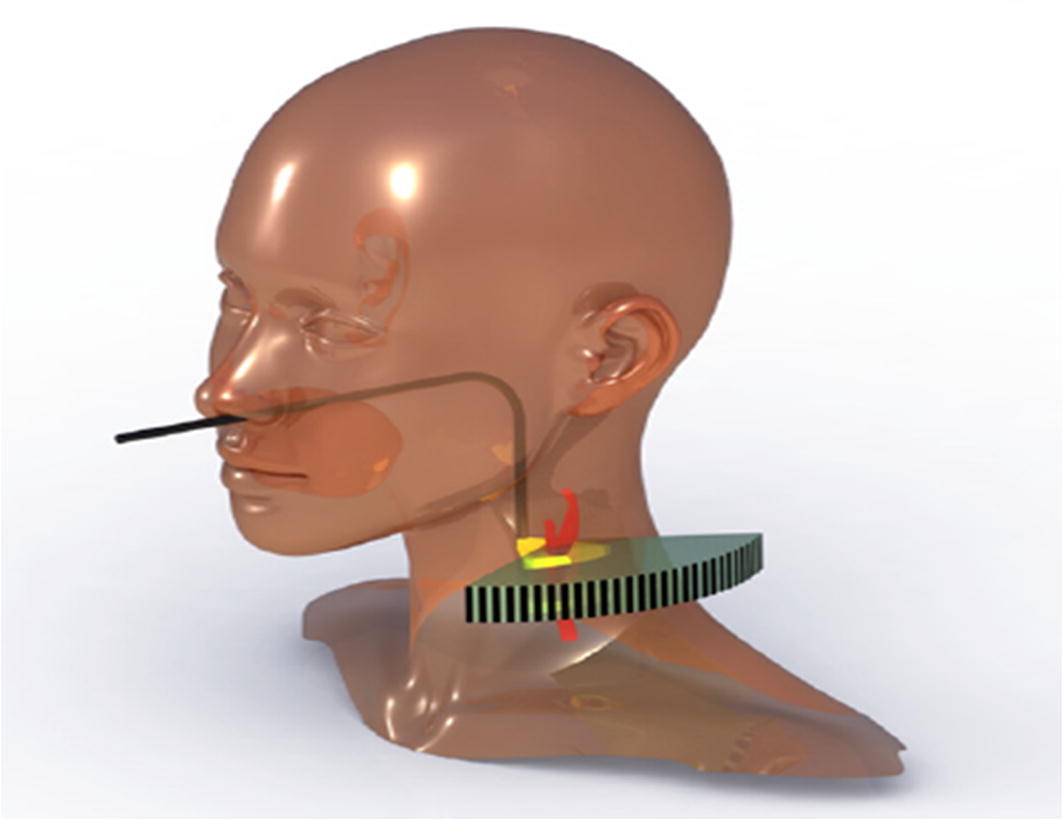
Proposed PAT method of internally illuminating the carotid artery through pharynx and detecting the acoustic signal from outside using concave transducer array [120]

Thermal expansion of the human tissue, upon application of a modulated electromagnetic energy, produces an acoustic wave [118]. The energy applied is selectively absorbed by the tissue depending on its absorption properties. This led to tissue expansion due to heating, resulting in an acoustic signal. Detecting these acoustic signals with the help of a transducer, an image is formed using back projection algorithm either in time or frequency domain. This is called photoacoustic tomography (PAT) or optoacoustic (OAT) or thermoacoustic tomography (TAT). The energy source could be a laser, microwaves, optical or radio frequency waves, etc. Application of multi-spectral OAT, using Nd:YAG laser, can be used to visualize the human carotid artery and jugular vein [119]. In an ex vivo study, validated histologically, molecular composition of the tissue (water, collagen, fatty acids, and plaque lipids) has been proven to be successfully identified using PAT [120]. Further, a possible method to measure and detect the lipid in carotid artery atherosclerosis, through internal illumination and signal detection from external side of the neck, is shown in Fig. 11. Based on the limited literature findings, a more intensive investigation in the field could realize a strong diagnosis tool in the near future.

#### Infrared (IR) thermography

Other than the physiological changes at the site of atherosclerosis, an acute inflammatory reaction also takes place at these sites of lesion [121], leading to a rise in the localized temperature [122]. In a histological study on 48 patients, an inverse correlation is found between the plaque fibrous cap thickness and plaque surface temperature [123]. Given that the carotid artery is obstructed (partly or completely), the effective blood perfusion to the facial and forehead skin tissues will be affected in comparison to a normal carotid artery blood flow. This in return affects the skin temperature in the region. In the past, usefulness of plate thermography, wherein a flexible foil coated with specific cholesterol esters that changes the foil colour due to temperature oscillations, was examined [124]. Applying the foil on the forehead of the patients, an indirect measure of the decrease in skin blood perfusion (decrease in temperature) was established. Out of 300 patients studied, plate thermography gives positive results (abnormality) in 39 patients against 34 patients using Doppler sonography. In all, both the methods agree for 23 patients, followed by false-positive and false-negative results in 5 and 3 patients, respectively [124]. To perform a noncontact temperature measurement, facial Infrared (IR) thermography, on 30 patients suffering from angiographically proven carotid stenosis or occlusion, was also used [125]. With a routine IR thermography exam, an abnormal thermal map was found in 57% of the cases as a possible sign of carotid disease. Further, a proactive thermography test using head clamp cooling can be used, which showed an increase of 83% in the sensitivity of the test [125]. In another study, the degree of stenosis was correlated to the ocular temperature on 24 patients ($r = - \,0.67$) [126]. Therefore, thermography stands a strong potential in diagnosing CAS. In future, an active thermography method, wherein external stimulation is done to extract maximum thermal features [127, 128] could be a potential advancement in the field.

## Conclusions

Accuracy of CAS diagnosis has substantially increased over the decades with the progressive technological developments. From the measurement of the narrowing carotid artery diameter to the evaluation of the increased velocity field near the obstruction/lesion site, in the carotid artery, increased emphasis has now been on the detection of additional parameters to characterize the plaque vulnerability. Use of computer-aided programs has further improved the sensitivity, specificity and accuracy of CAS diagnosis through various imaging modalities. DSA is the gold standard to diagnose CAS but suffers from the X-ray radiation exposure drawback. With optimized X-ray radiation, CTA provides 3D visualization, but is inefficient in evaluating the blood flow velocity. MRA is another method, with high accuracy and resolution, which eases the characterization of vulnerable plaque, but the equipment cost is high and not readily available everywhere. DUS is of low cost, but its efficacy is operator dependent. Evolution of computer-based diagnosis algorithms and 3D ultrasound systems can potentially address these issues. Intravenous ultrasound contrast brings substantial improvement to the overall performance of DUS. In the parallel imaging technological development, potential imaging modalities, like OCT, PAT, and Thermography, are also evolving over time. Through multiple in vivo human subject studies, OCT has already been proven to be a strong potential alternative. Unlike conventional imaging modalities, OCT can detect plaque characteristics more efficiently. Thermography shows a strong diagnosis potential through a correlation between stenosis and ocular temperature. Although successfully studied in ex vivo models, PAT must go a long way in the field of CAS diagnosis.

## Data Availability

Not applicable.

## Abbreviations

CVD: cardiovascular disease
CAS: carotid artery stenosis
CAD: coronary artery disease
DUS: duplex ultrasound
IMT: intima media thickness
BM: block matching
ROI: region of interest
DE: Doppler effect
DS: Doppler shift
CWD: continuous-wave Doppler
PWD: pulsed-wave Doppler
DSA: digital subtraction angiography
CTA: computed tomography angiography
MRI: magnetic resonance imaging
MRA: magnetic resonance angiography
OCT: optical coherence tomography
PAT: photoacoustic tomography
